# Hybrid PCA–LBP and Wavelet Scattering Framework for Texture Classification in Color Images

**DOI:** 10.3390/jimaging12080372

**Published:** 2026-08-11

**Authors:** Zahoor M. Aydam, Baidaa Mutasher Rashed, Nidhal K. El Abbadi

**Affiliations:** 1 Department of Information Technology, College of Computer Science and Mathematics, University of Thi-Qar, Nasiriyah 64001, Iraq; 2 Department of Computers, Faculty of Computer Science & Mathematics, University of Thi-Qar, Nasiriyah 00964, Iraq; baidaaalsafy@utq.edu.iq; 3 Engineering College, University of Cordoba, Najaf 54001, Iraq; nidhal.abass@fulbrightmail.org

**Keywords:** XGBoost classifier, pattern recognition, image feature extraction, machine learning image processing, computer vision

## Abstract

Color texture classification is an important task in computer vision, with applications in medical imaging, industrial inspection, remote sensing, and material analysis. This paper presents a hybrid framework that integrates Principal Component Analysis (PCA), Local Binary Patterns (LBPs), Wavelet Scattering Transform, and the XGBoost classifier for color texture classification. The proposed pipeline first performs image pre-processing, including resizing and denoising, followed by channel-wise feature extraction using LBP and Wavelet Scattering Transform on the Red, Green, and Blue channels independently. Then, the obtained feature vectors were concatenated, and PCA was applied on the fused feature space for dimensionality reduction and redundancy elimination before proceeding to XGBoost classification. This method not only leverages complementary information of Chroma and texture information but also achieves reduced dimensionality and computational burden. The finally optimized features were input into the XGBoost classifier for color texture classification, which is good at fitting non-linear dependency and includes a regularization to generalize better. Our proposed framework was tested on three benchmark color texture datasets: KTH-TIPS, Outex_10, and VisTex. Experimental results have demonstrated that on these three datasets, the average performance reaches 98.0% accuracy, 0.981 precision, 0.981 recall, and 0.979 F1-score, respectively. It demonstrates that the two selected complementary feature extraction methods provide a compact yet effective representation for color texture classification on these datasets. It is expected that the proposed framework serves as an efficient combination of established methods and as a good competitive baseline for color texture analysis. Future works will consider applying it to larger color texture datasets for general verification, enhancing its computational efficiency and automating the parameter selection process.

## 1. Introduction

Texture information is widely used in image analysis techniques, feature extraction, and content interpretation. Texture can be seen as the spatial variation of pixel intensity and is a key feature for a number of applications; for instance, it is used in medical imaging analysis, content-based image retrieval, remote sensing images, and object identification and classification. There is no exact definition for texture, but it can generally be described as the features of coarseness, regularity, smoothness, granularity, and randomness [1]. Texture analysis has always been a difficult area, especially in content-based systems, since it is affected by lighting, viewpoint, and surface deformities.

Texture classification usually consists of two main stages: feature extraction and classification. Feature extraction has been a key area of research in texture classification due to the importance of good and discriminant feature representation for classification. Extensive work has been conducted on developing compact and robust texture descriptors to model repetitive structures and local variations in illumination across scenes.

However, the performance of texture description and classification is sensitive to various factors such as illumination changes, rotation, occlusion, and noise [2].

Specifically, one of the most significant difficulties is color features, which are very sensitive to changes in illumination and acquisition conditions [2].

The success of Convolutional Neural Networks (CNNs) and Vision Transformers (ViTs) has inspired many research works in the usage of deep learning methods for texture classification, thanks to the inherent learning of hierarchical feature representations for visual features. Unfortunately, training such models requires huge datasets and massive computational resources. Alternatively, handcrafted and math-based lightweight and interpretable methods provide competitive performances, which can be advantageous in some real-world application cases.

Moreover, textural datasets are characterized by high intra-class variations and low inter-class separation, which result in lower classification performance. In such contexts, texture descriptors have to be able to capture the discrimination between different classes in a computationally efficient manner. Local Binary Patterns (LBPs) [1,3] have become one of the most popular texture feature descriptors due to their simplicity, robustness to monotonic gray-scale transformation, and invariance to gray-scale transformation [1]. Originally proposed for gray-scale images, LBP can also be used for multi-channel and color images, and then it can be applied to color texture classification [4].

However, most handcrafted texture descriptors are subject to some problems, including redundant features, sensitivity to noise, and low tolerance for geometric and illumination variations, when applied alone. Although PCA, LBP, WST, and XGBoost have independently shown good performance in texture description tasks, the co-utilization of these features for color texture classification has not been deeply researched.

Here, to fill this research gap, we propose a hierarchical feature integration framework for addressing such problems. In the beginning, the input RGB image is divided into Red (R), Green (G), and Blue (B) channels. Then, the LBP feature is extracted for each channel independently in order to maintain distinct channel information (chromatic and structural). Secondly, applying Wavelet Scattering Transform to each channel-wise LBP feature can capture robust and stable multi-scale features against small deformations and translations.

Third, the features of each channel are concatenated, followed by PCA feature dimension reduction so that the redundant information can be deleted and the most discriminative color texture properties can be reserved. Lastly, XGBoost is applied to classify the final optimal features, which is better for classification without overfitting. Furthermore, utilizing XGBoost not only greatly increases feature discrimination ability but also leverages the efficient computation and good generalization of XGBoost against noisy data and high-dimensional features. Finally, we evaluate the proposed approach on multiple well-known datasets including KTH-TIPS, Outex_10, and VisTex under challenging and noisy conditions. Our motivation is to resolve issues regarding illumination variations affecting color texture features and the insufficient data of available classification methods.

The main contributions of this work can be summarized as follows:

- We proposed a novel hybrid framework for color texture classification by jointly using channel-wise Local Binary Patterns (LBPs), Wavelet Scattering Transform (WST), Principal Component Analysis (PCA), and XGBoost in an organized, structured pipeline.
- We proposed a concise and effective channel-wise extraction method to retain rich discriminatory colors before reduction, in contrast to typical PCA-based texture methods that lose visual texture structures.
- We modeled discriminative representations using multi-scale information through the union of local texture features with wavelet scattering, capturing varied textures regardless of illumination variation, scale, and noise.
- We conceived a classification pipeline immune to the overfitting problem, being well-suited for small datasets and deep networks (which has difficulties in generalization) when the amount of data is low.
- We perform a full range of empirical studies, including k-cross-validation, ablation studies, statistical evaluations, and robustness to noise using the well-established KTH-TIPS, Outex, and VisTex datasets.
- In experimental evaluations conducted on the most frequently used benchmark texture datasets, we achieved a classification accuracy of 98.01%, outperforming existing deep learning approaches under exactly the same experimental conditions, with superior computational efficiency and good interpretability.

## 2. Related Works

Texture classification has received great attention in many applications of computer vision, medical imaging, industrial inspection, remote sensing, and material recognition. Generally, the current methods can be systematically categorized into three groups: (1) handcrafted single descriptors, (2) multi-scale enhanced handcrafted descriptors, and (3) deep learning-based methods. Different groups have their advantages and disadvantages in terms of descriptive capability, complexity, robustness, and interpretability.

### 2.1. Handcrafted Single Descriptors

Historically, image texture classification schemes utilize handcrafted features and descriptors, e.g., Local Binary Patterns (LBPs) [4] and Gabor filters [2,3]. The primary reasons are high computational efficiency and the ease of feature interpretation for the resulting classification model, which are important to applications in real-time and embedded devices.

Hosny et al. [5] merged CNN features and LBP in conjunction with an SVM classifier for color texture recognition.

The empirical results confirmed the fact that LBP improves discriminative capabilities. Luimstra and Bunte [6] developed an adaptable Gabor filter-based scheme that enabled visual interpretable color textures by keeping the number of learnable parameters to a minimum.

Limitations: Disadvantages: Single handcrafted descriptor. Although simple, single handcrafted descriptors lack representational power since they just consider micro-patterns at the local position and cannot model the complicated multi-scale texture. They are also poor in noise, illumination change, and geometry variation robustness.

### 2.2. Multi-Scale Improved Handcrafted Descriptors

To circumvent drawbacks of single-scale basic descriptors, there are studies to develop multi-scale and noise-robust extended LBP algorithms. For example, Rong et al. [7] proposed the Multiscale Group Variance Binary Pattern (MGVBP), which includes information on local groups and their variance to achieve high robustness under noise effects. Luo et al. [8] proposed Scale-Selective Noise-Robust Extended LBP (SNELBP), combining Gaussian scale-space theory and the compact feature selection technique to achieve improved scale invariance and robustness to noise effects.

Limitations: Disadvantages: These approaches, while providing higher robustness and discriminative ability, tend to impose higher computational burdens owing to multi-scale and high-dimensional features. Their effectiveness at detecting hierarchical and global texture is still insufficient.

### 2.3. Deep Learning-Based Texture Classification

Nowadays, with the breakthrough of deep learning approaches such as Convolutional Neural Network (CNN), Vision Transformer (ViT), and Swin Transformer, texture classification has reached record-breaking performance due to the representations and hierarchies learned directly from the data.

Limitations: However, the requirements for large-scale labeled training data, high computational resources, and long training time are the shortcomings for training deep learning models, and these models do not have clear explainability due to their black-box nature in applications such as image recognition or medical inspection.

### 2.4. Hybrid and Color Texture Representation Methods

Beyond these kinds of approaches, there were some studies on hybrid models, color texture models, and so on. Shumska et al. [9] presented a framework for learning a frame GMLVQ approach that can achieve spatial color representation by the use of limited training examples and guarantee good interpretability. Fares and Ribas [10] have proposed Volumetric Color Texture Representation (VCTex) that uses 3D color cubes in the auto-encoder association in order to represent spatial and spectral features.

Limitations: These methods are effective for representation ability but sometimes have the issue of weak generalization ability to varying illumination or poor robustness to strong noise.

### 2.5. Research Gaps and Motivation

There are several limitations in prior work, although the methods were quite good. There was not enough stability for multi-scale representation by handcrafted descriptors. The new types were more complicated but still not fully sufficient in hierarchical feature extraction, and deep learning methods required vast training datasets and computing power. In many cases, the methods require either a huge dataset of textures or much more computing power, which may not be possible when few texture samples are available for learning.

Also, less has been done in incorporating dimensionality reduction, local texture feature extraction, multi-scale wavelet scattering, and classification based on a strong learning algorithm in a single model with proper interpretabilities.

So, we proposed a model that integrates PCA with Local Binary Patterns, Wavelet Scattering Transform, and XGBoost classification to obtain discriminative, compact, multi-scale, and computationally efficient texture feature descriptors and classifiers.

## 3. Problem Statement

One of the primary problems in digital image processing is texture analysis and point of interest in digital image processing, which have significant applications in medicine, space surveillance, and industry. In spite of all the existing methods, however, they are still limited in terms of accuracy, generalization, and stability when exposed to changes, including scaling, rotation, and light. Hence, the necessity to introduce new methodologies that provide greater accuracy and generalizability is evident, as it will guarantee the successful analysis and enhance the results of real-life applications.

## 4. Local Binary Pattern (LBP)

**Figure 1:**
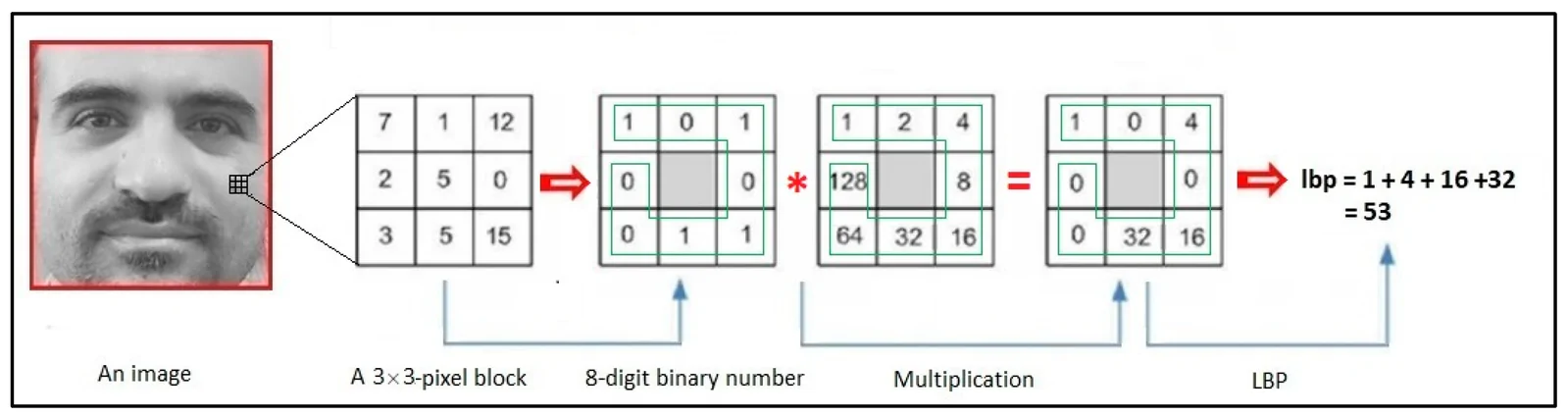
LBP value computation process. Each 3 × 3-pixel block in the image is encoded to an LBP value [12].

Local Binary Pattern (LBP) is a widely used texture descriptor in computer vision, particularly effective at capturing the spatial structure of local image regions. The LBP operator encodes texture by thresholding a neighborhood of pixels based on the intensity of the central pixel. The image is first divided into a set of local blocks, and within each block, the LBP code is computed for each pixel. As shown in Figure 1, the standard LBP operator considers a 3 × 3 neighborhood around each pixel [11]. LBP is calculated according to the rule in Equation (1).

(1) $$s\left(p,c\right)=\left\{\begin{array}{c} 1\;\;\;\;\;\;\;\;\;\;\;\;g(p)\geq g(c)\\ 0\;\;\;\;\;\;\;\;\;\;\;\;\;\mathrm{otherwise} \end{array}\right.$$

where g(c) is the intensity value of the central pixel and g(p) is the intensity of one of its eight surrounding pixels.

By applying this rule to all eight neighbors $p_{1},p_{2},\ldots ,{\;p}_{8}$, the binary outcomes are weighted and summed to generate the Local Binary Pattern (LBP) value at pixel *c* as follows:

(2) $$lap(c)=\sum_{p=1}^{8}2^{p-1}S\left(p_{i}-c\right)$$

## 5. Principal Component Analysis (PCA)

Principal Component Analysis (PCA) is a widely used statistical technique for dimensionality reduction, particularly effective at analyzing large-scale datasets. It transforms the data into a new coordinate system where the greatest variance by any projection of the data lies on the first coordinate (principal component), the second greatest variance on the second coordinate, and so on. This allows for the identification of significant patterns, such as distinguishing tumor regions in medical imaging, by preserving the maximum variance in the data [13].

## 6. Wavelet Scattering Network

Wavelet scattering is a powerful mathematical representation of image feature extraction and analysis; it provides an intuitive method of describing textures by taking images into the wavelet domain. Wavelet Scattering Transform is an effective signal processing model, which is resistant to translations and can be performed with small deformations, and this property makes it especially popular in classification tasks. This technique involves cascading traditional wavelet transforms with a non-linear modulus and averaging filters in order to restore the high-frequency information that is normally lost at the first stage of the wavelet decomposition phase [14].

**Figure 2:**
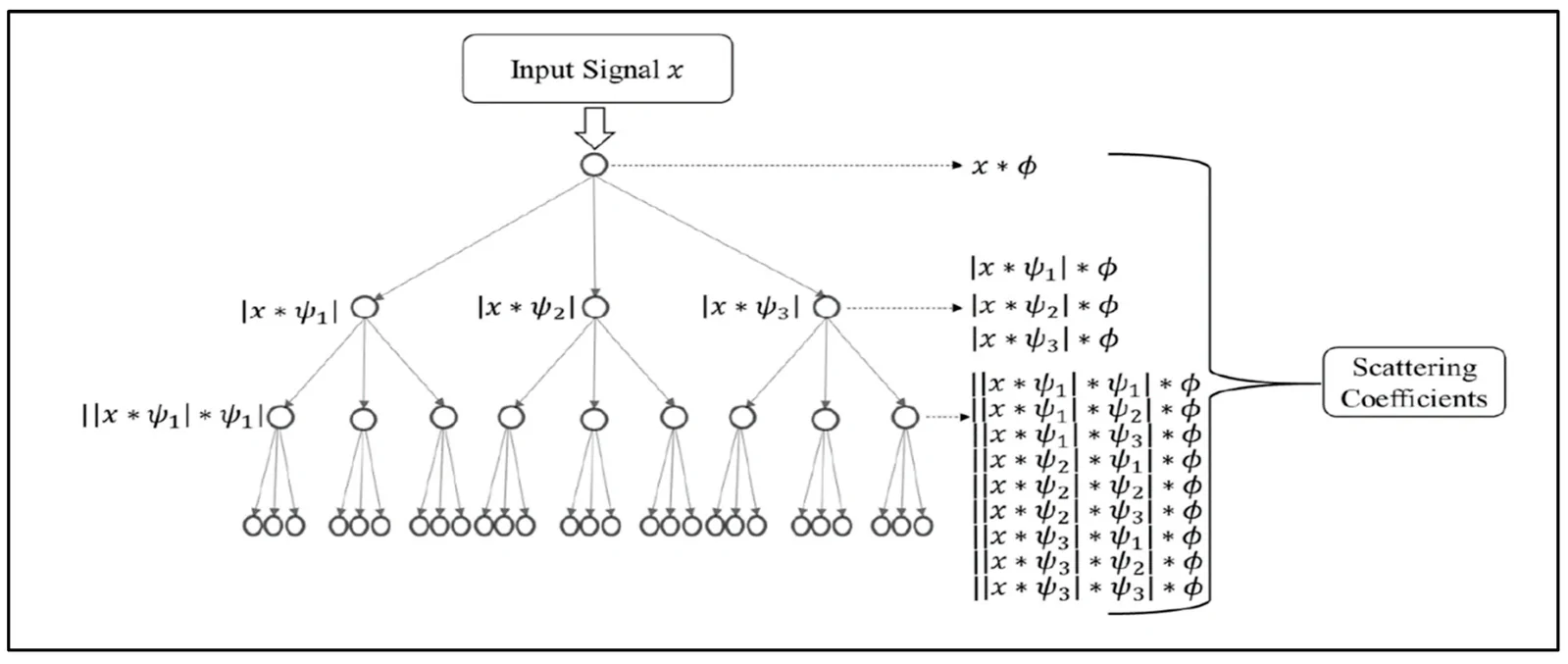
Wavelet Scattering Network [15].

The hierarchical decomposition involved in wavelet scattering is illustrated in Figure 2.

### Wavelet Scattering Hierarchical Features

The main idea is to subdivide a signal into the consecutive scattering orders, each order containing various degrees of structural information, which is stable to deformations and invariant to translations [16].

Zero-order wavelet scattering coefficients are the initial signal, which has been mixed with a low-pass filter, and are used as a starting point in further transformations. This order preserves the global, low-frequency components of the signal but does not add any structural features. As shown in Equation (3), the zero-order coefficient $S_{0}$ is defined as:

(3) $$S_{0}=x*\emptyset$$

where *x* is the input signal (or image) and *ϕ* is a low-pass filter. The coefficients give translation-invariant descriptors that are used to provide reference features that are used to compare and normalize in further analysis [17,18].

First-order wavelet scattering coefficients represent localized spatial characteristics of an input signal through the convolution of an input signal with a filter family of wavelets. Such attributes represent the major content of the signal-like edges and textures that are critical in classification activities. The coefficients are calculated by the calculation of the modulus of the wavelet-transformed signal and averaged with the use of the same low-pass filter. The scattering coefficients of the first-order S_1_ are written down mathematically as:

(4) $$S_{1}=|x*\psi_{\lambda 1}|*\emptyset$$

in which $\psi_{\lambda 1}$ will be a complex or real-valued wavelet filter (e.g., Morlet, Haar, or Gabor) at scale *λ*_1_, and the modulus operator will guarantee non-linearity and preserve essential amplitude information [19,20].

Second-order wavelet scattering coefficients are an extension of the analysis of the interaction of two frequency components and show more complex and higher-order structures of the signal. These coefficients have excellent ability to capture complex textures and non-local dependencies that cannot be seen at the first order. The calculation entails repeated conviction and modulus operations for the first-order transformed signal, and then averaging with the low-pass filter. The second-order coefficients S_2_ are given by:

(5) $$S_{2}=|\left|x*\psi_{\lambda 1}\right|*\psi_{\lambda 2}|*\emptyset$$

in which the wavelet functions at various scales *λ*_1_ and *λ*_2_ are denoted by $\psi_{\lambda 1}$ and $\psi_{\lambda 2}$, respectively. This recursive structure allows for the modeling of higher-order dependencies in various resolutions [21,22].

## 7. Extreme Gradient Boosting (XGBoost)

XGBoost is a more complicated version of the Gradient Boosting Machine (GBM), which in turn is a combination of gradient descent and boosting. Boosting is an ensemble strategy learning, which re-allocates training data by using different weights for each training sample at each step. Here, incorrectly classified samples are given more weight, and correctly classified samples are given less weight, effectively focusing the model on more difficult-to-classify samples and improving the overall predictive accuracy [23]. The GBM is an optimization technique that makes use of second-order gradient data to solve a regularized purpose function, as shown in Equation (6).

(6) $$l\left(\varphi \right)=\sum_{i}I\left({\hat{y}}_{i},y_{i}\right)+\sum_{k}\Omega (f_{k})$$

(7) $$\Omega \left(f_{k}\right)=\gamma^{T}+\frac{1}{2}\gamma {\left|\left|w\right|\right|}^{2}$$

where *I* represents a differentiable convex loss that measures the difference between the predicted value ${\hat{y}}_{i}$ and the true target *y_i_*, and Ω represents a regularization term that penalizes model simplicity in order to prevent overfitting [24]. The GBM is a tree-based learning algorithm. It involves the identification of optimal split points in decision trees, which is computationally expensive when dealing with large datasets. To overcome this difficulty, XGBoost proposes a new distributed, weighted quantile sketch algorithm that can efficiently work with weighted data. This algorithm has solid theoretical justifications and allows for computing split candidates efficiently and at scale, thereby substantially improving the training efficiency and accuracy of the boosting process.

## 8. Performance Matrices

The performance of texture classification models is evaluated using a confusion matrix. It visualizes an algorithm’s performance through visualizing TP, TN, FP, and FN. Performance evaluation metrics are also gauged in this way, which gives additional information regarding the model rather than accuracy in the general sense [3]. Precision, recall, and F1-score are primarily crucial metrics for texture classification.

- Precision is the number of accurate positive observations over the total number of predicted positives; it can be defined as [25]:
  (8) $$Precision=\frac{TP}{TP+FP}\;\;\;\;\;$$

- Recall is the number of accurate positive observations over the total number of observations in the actual class; it can be defined as [26]:
  (9) $$Recall=\frac{TP}{TP+FN}\;$$

- F1-score is the precision and recall harmonic mean; it can be defined in Equation (10) [27]:
  (10) $$F1-Score=2\times \frac{Precision\times Recall}{Precision+Recall}\;$$
  where TP is True Positive, TN is True Negative, FP is False Positive, and FN is False Negative.

## 9. Proposed Model

**Figure 3:**
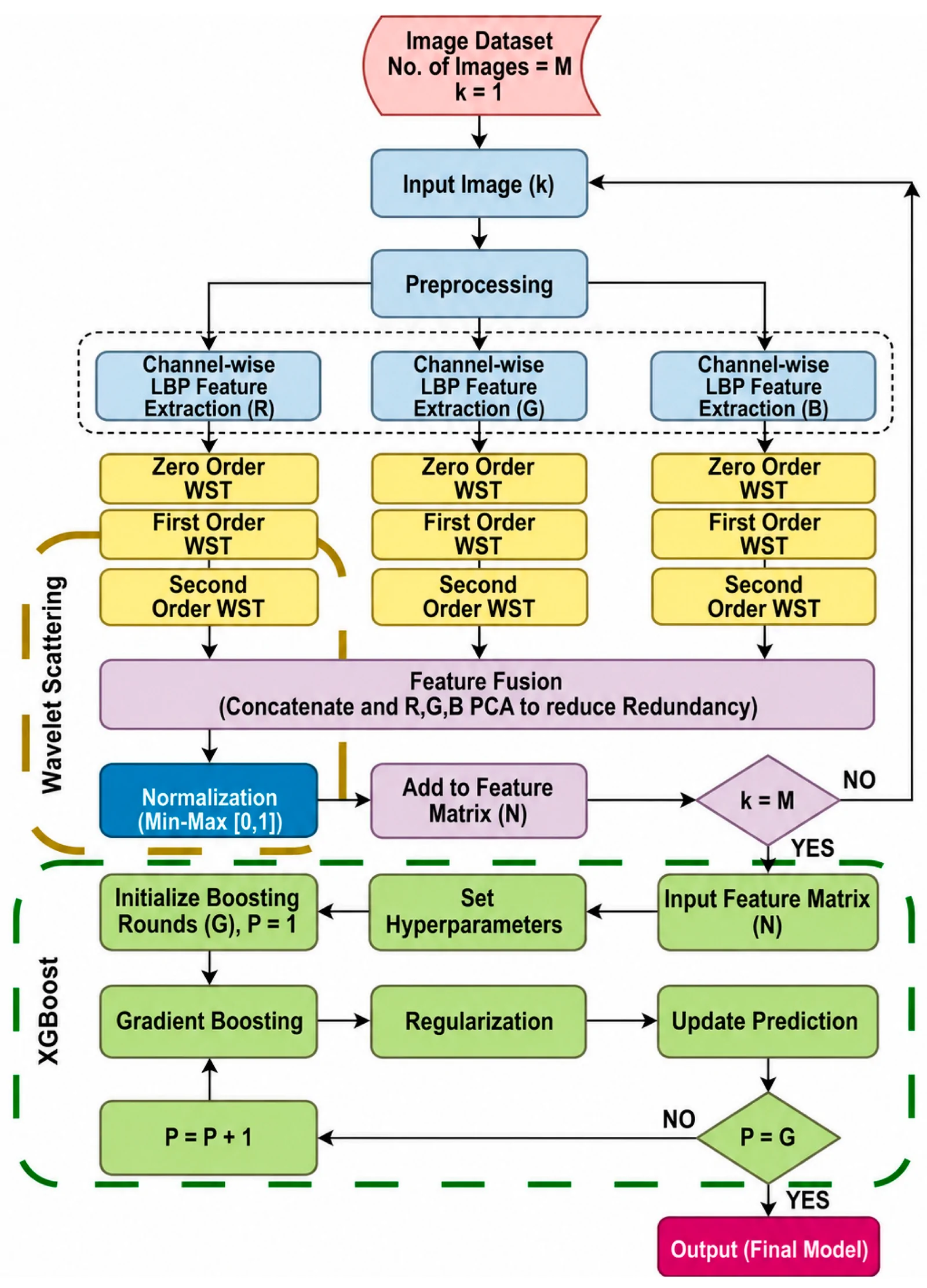
Flowchart of the proposed model.

The proposed model, described in this section, comprises five stages: in the first stage, selected texture datasets are used as inputs for analysis in subsequent steps; in the second stage, pre-processing of the datasets is performed; in the third stage, feature extraction is performed to aid classification using wavelet scattering; in the fourth stage, features are classified using the XGBoost classifier; and finally, the proposed model is validated using performance metrics (precision, recall, and F1-score). Figure 3 shows the overall framework of the proposed method. Our model leverages PCA–LBP, wavelet scattering, and XGBoost classifier for improved texture classification because PCA–LBP, wavelet scattering, and XGBoost offer stable and meaningful feature representation. To mitigate the limitations of traditional texture features, i.e., the susceptibility of color features to varying lighting conditions, the PCA–LBP–Wavelet Scattering Framework employs PCA for minimizing the redundancy between the channels in color images and for enhancing robustness to illumination.

In addition, wavelet scattering is used for its capacity to extract discriminative features in the presence of minor illumination and geometric distortion, and LBP can also capture the finer details of local textures. PCA reduces the irrelevance between color channels and makes the features more discriminative and less affected by the illumination.

This work proposes a computational efficiency and accuracy model for the classification of color textures by utilizing Principal Component Analysis (PCA), Local Binary Patterns (LBPs), and Wavelet Scattering Transform (WST), with XGBoost as a classifier. The primary contributions are as follows:

1. An effective framework with a novel approach for the extraction of hybrid color texture features, in which PCA helps to extract decorrelated colors, and LBP and WST extract local and multi-scale textural features, respectively.
2. A dimensionality reduction approach to extract the dominant information and reduce computation in order to enhance classification performance.
3. A comprehensive experimental evaluation using multiple benchmark datasets under both hold-out and cross-validation protocols.
4. An ablation study that quantitatively demonstrates the contribution of each component within the proposed pipeline.
5. A computational analysis that evaluates the efficiency and practical feasibility of the proposed approach.

Training stage: aims at preparing a model that will be able to classify texture, regardless of whether it is an image that can be seen or not. For even one unseen image, processing steps may be proposed.

Step 1: Image Pre-processing

The very first step in the proposed framework deals with the pre-processing of real images so as to be ready for image analysis. A summary of the primary process steps performed at this stage is shown in Table 1.

Step 2: Channel-wise Color Representation

In order to mitigate potential information loss due to dimensionality reduction (due to potentially less information content in reduced space), a channel-wise processing approach is utilized in our proposed framework. Rather than reducing the RGB image into a single intensity, or directly conducting PCA on the raw color image, we first decompose the input image into its respective R, G, and B components.

Each color channel is then independently treated with the intention of retaining the channel-specific texture variation, as well as the color distribution characteristics of the colors for the purpose of reliable color texture classification.

Subsequently, we independently calculate Local Binary Pattern (LBP) for each color channel, thus retaining chromatic and structural details within each component, then concatenate them, producing a composite color-aware texture descriptor. Following the calculation of such feature vectors, we then use PCA to reduce any resultant feature redundancy in the concatenated feature vector space. Doing so prevents the suppression of color features before extraction and balances complementary features such as color and structure throughout feature extraction. PCA was used since it is a very commonly utilized linear dimensionality-reducing algorithm; it finds the largest possible variance in the data while removing any features that are heavily correlated. Unlike using other color space transformations like HSV, CIELab, or YCbCr for the task of capturing color information in a specific perception space, we apply PCA to optimize the features after our channel-wise processing and extraction.

Reducing the dimensions of the combined vector using PCA helps us make computational analysis faster and efficient, prevent overfitting, and retain the maximum amount of descriptive color information extracted in individual channel analyses.

In addition, we employ the Wavelet Scattering Transform to the multicomponents of the computed representation to extract stable and multi-scale feature representation robust to distortions and deformations but preserve sensitive color details. Our complementary utilization of channel-wise LBP, Wavelet Scattering Transform, PCA, and XGBoost gives us a compact and effective color texture classifier. Overall, channel-wise treatment serves to effectively minimize early color information loss and strengthen the discriminatory power, efficiency, and robustness of our model for color texture classification.

Step 3: Channel-wise LBP Feature Extraction

We apply the LBP independently on each RGB channel of the image in order to extract the local texture descriptor such as edge, spot, and flat areas. Thus, fine-grained texture description of each color channel can be extracted individually. The LBP feature maps derived from the Red, Green, and Blue channels are thus retained and will be further processed so that they simultaneously preserve the spatial and chromatic texture description.

Step 4: Normalization

To prevent large numbers from dominating other numbers and to prevent the outlier effect, in addition to balancing contributions, numbers are normalized to the [0, 1] range. In this article, data are normalized using Min–Max normalization to ensure that numbers have a stable distribution, then these data are used for wavelet scattering and classification.

Step 5: Wavelet Scattering Transform for Channel-wise Features

We compute the second-order Wavelet Scattering Transform (WST) for each LBP-transformed color channel individually. This can isolate the stable multi-scale and orientation-selective features per channel. Combining WST from all channels reveals hierarchical representations for textures integrating both structure and color differences. In the last step, we concatenate all scattering coefficients from each channel and any desired order.

Step 6: Feature Fusion

The feature vectors from the three channels are stacked one on top of the other to create a single feature vector from each image. The matrix generated in this way has an image per row, and the columns contain features from fused descriptors based on multichannel texture information. This procedure increases the distinctiveness of the feature set because it combines the discriminative aspects of each color channel separately.

Step 7: Classification Using Extreme Gradient Boosting Classifier

The feature vectors computed during the steps above were classified by an Extreme Gradient Boosting (XGBoost) classifier. XGBoost was used because the algorithm effectively captures non-linear patterns, supports high-dimensional feature vectors, and its regularization parameters can control overfitting without losing model prediction ability. The classifier parameters have been adjusted using heuristic-determined hyperparameters.

Specifically, n estimators (number of boosting rounds to run) are set to 300, learning rate (shrinkage rate) to 0.05, max depth of tree (maximum depth to build tree) to 6, subsample ratio of training instances to consider for each tree (the fraction of instances) to 0.80, feature sampling ratio of columns for each tree to consider (the fraction of columns to consider) to 0.80, min child weight to 1, and gamma (minimum loss reduction to be incurred to make a split on a leaf node) is set to 0.

We used the multiclass objective (multi:softprob) for prediction and set a consistent random seed (42) across experiments. We determined these hyperparameters by a preliminary set of empirical experiments where we tried various parameters and evaluated model performance based on training performance. The training of XGBoost can be described by the following process: In each round i (from 1 to T), a new decision tree T_i_ is fitted to the residuals of tree (T_1_, …, T_i−1_), namely T_i_ = $\underset{T}{\arg\min\;L}\left(y,F_{I-1}(x)+\alpha T(x)\right)$, where α is the step size. Each new tree minimizes the residual errors of the previous tree ensemble using first- and second-order gradient information of the loss function L (y, F(x)).

Model complexity is regularized by using L1 and L2 penalties, which help avoid over-fitting and increase generalization performance.

L1 regularization is formulated as

(11) $${\left||w|\right|}_{1}=\sum \;|\;w_{i\;}|$$

whereas the L2 regularization term is expressed as

(12) $${{\left||w|\right|}^{2}}_{2}=\cdots \;{\;||\;w}_{2\;}^{2}||=\sum w_{i}^{2}$$

where $w_{i}$ represents the importance of the model’s parameters.

Testing phase: During the testing phase, the trained model is tested with few unused images. Again, the same procedure from steps 1–6 is followed for creating the feature vector.

The model is tested not only on clean images but also with the help of adding Gaussian and salt-and-pepper noises to degrade the images and check how the framework performed.

Again, the generated features are passed to the trained XGBoost classifier for determining texture classification.

### Implementation Details

All experiments were performed on an Intel(R) Core(TM) i7-7700k CPU @ 3.40 GHz, 32 GB RAM workstation, running on Windows 11 (64-bit). A hybrid implementation has been chosen in order to use each of the frameworks to perform parts of the tasks for which it is best fitted: Image pre-processing, Principal Component Analysis (PCA), Local Binary Patterns (LBPs), and Wavelet Scattering Transform (WST) were computed using MATLAB R2024b whereas the XGBoost classifier was implemented in Python 3.11 with standard libraries (NumPy, Scikit-learn, XGBoost). In order to make the results completely reproducible, a fixed random seed was always chosen (seed = 42) throughout the entire process (splitting of the data, model training, etc.).

Data Pre-processing and Feature Extraction

The images were first resized to the same size of 256 × 256 to have the same input in both datasets for feature extraction, and Min–Max normalization is used to normalize each pixel value to [0, 1] to decrease the effect of the illumination variation. The image of each channel (R, G, B) was analyzed individually in order to preserve the discriminative information present in the colors, and PCA, LBP, and WST were combined to extract the features:

- PCA Configuration:

Number of components: selected to retain ≥95% variance

Whitening: disabled

Solver: auto

- LBP Configuration:

Radius: R = 1

Number of neighbors: P = 8

Method: uniform

Output: histogram-based feature vector

- Wavelet Scattering Transform (WST):

Wavelet type: Morlet

Number of layers: 2

Number of orientations: 8

Invariance scale: predefined (default MATLAB setting)

We then concatenated each channel to build a unique feature vector which is reduced with PCA.

Classification and Model Configuration

For classification tasks, we utilize the XGBoost model because of its effectiveness and strong performance in modeling with structured feature representation.

- XGBoost Hyperparameters:

Learning rate: 0.1

Number of estimators: 100

Maximum tree depth: 6

Subsample: 0.8

Column sample by tree: 0.8

Objective: multi: softprob

Evaluation metric: mlogloss

Random seed: 42

The same hyperparameter configuration was used for each dataset to provide fair and unbiased results. K-fold cross-validation with k = 5 (stratified for class distribution balance) was used.

Deep Learning Baseline (CNN Configuration)

To provide a fair comparison with deep learning approaches, a simple Convolutional Neural Network (CNN) was implemented as a baseline model.

- CNN Architecture:

3 Convolutional layers (filters: 32, 64, 128)

Kernel size: 3 × 3

Activation: ReLU

Max pooling: 2 × 2

Fully connected layer: 128 neurons

Output layer: Softmax

- Training Parameters:

Optimizer: Adam

Learning rate: 0.001

Batch size: 32

Number of epochs: 25

Loss function: categorical cross-entropy

CNN models are trained and validated using the same split datasets, for fair comparison with our proposed method.

Computational Efficiency

With regard to performance, our framework achieved on average 14 ms per image inference time for an offline application using CPU, showing its practicality for offline use. In cases where real time is desired with low latency, parallelism with other processor architectures such as the CPU would be beneficial for further speeding up computation in GPUs.

## 10. Experimental Results

Outex_10, KTH-TIPS, and VisTex datasets were chosen because they have already become standard benchmark datasets in texture analysis-related research. They are characterized by the abundance of diverse realistic conditions, such as varying illumination, scales, viewing angles, color variations, and texture patterns. Thus, they offer a demanding and relevant benchmark environment to test the framework in its robustness, accuracy, and generalization potential under such a variety of challenging conditions. The statistical validation results of the obtained experimental data were further used for statistical analysis so that the reliability of our results could be further ensured. The results mentioned in this paper are the average of the obtained values over several experiments. Along with these evaluations, several more metrics were added for the robust evaluation of the classification model, such as accuracy, precision, recall, F1-score, etc.

In total, 5301 color texture images were selected in this paper to test the performance of our proposed framework.

The selected textures are a set of patterns, covering numerous classes of texture types, that can be defined in terms of their physical properties and morphology as follows: homogeneous (i.e., textiles, sand), directional (i.e., wood grains), and stochastic (i.e., plants, rocks).

These classes differ in spatial frequency, orientation, and the amount of their structural regularity and roughness and therefore constitute a comprehensive challenge. However, diversity is associated with classes that suffer more or less from a lack of training examples. Especially in the VisTex database, some texture classes only contain as few as nine examples, which leads to a higher risk of overfitting and reduced generalization of a model in data-driven methods. This problem applies to several of the common small-scale texture datasets.

The cross-validation approach combined with XGBoost classifier regularization features have been proposed to prevent the impact of class imbalance. The method for cross-validation ensures that every observation belongs to each testing dataset in one way or another, but given the small sample size of classes, we are still suffering from low generalizability performance. In addition, for the experimental setup, we split the data into train and test (85% training/15% test), but we take into account class preservation and five-fold cross-validation, so we consider the five-fold average to be a better performance estimate.

Also, pre-processing steps for the image sets have been applied equally across datasets. All images have been normalized with the same size, type, and process in terms of pixels; labels are confirmed for accuracy. Also, image normalization has been performed to consider the effect of lighting and acquisition system variation on image appearance.

**Figure 4:**
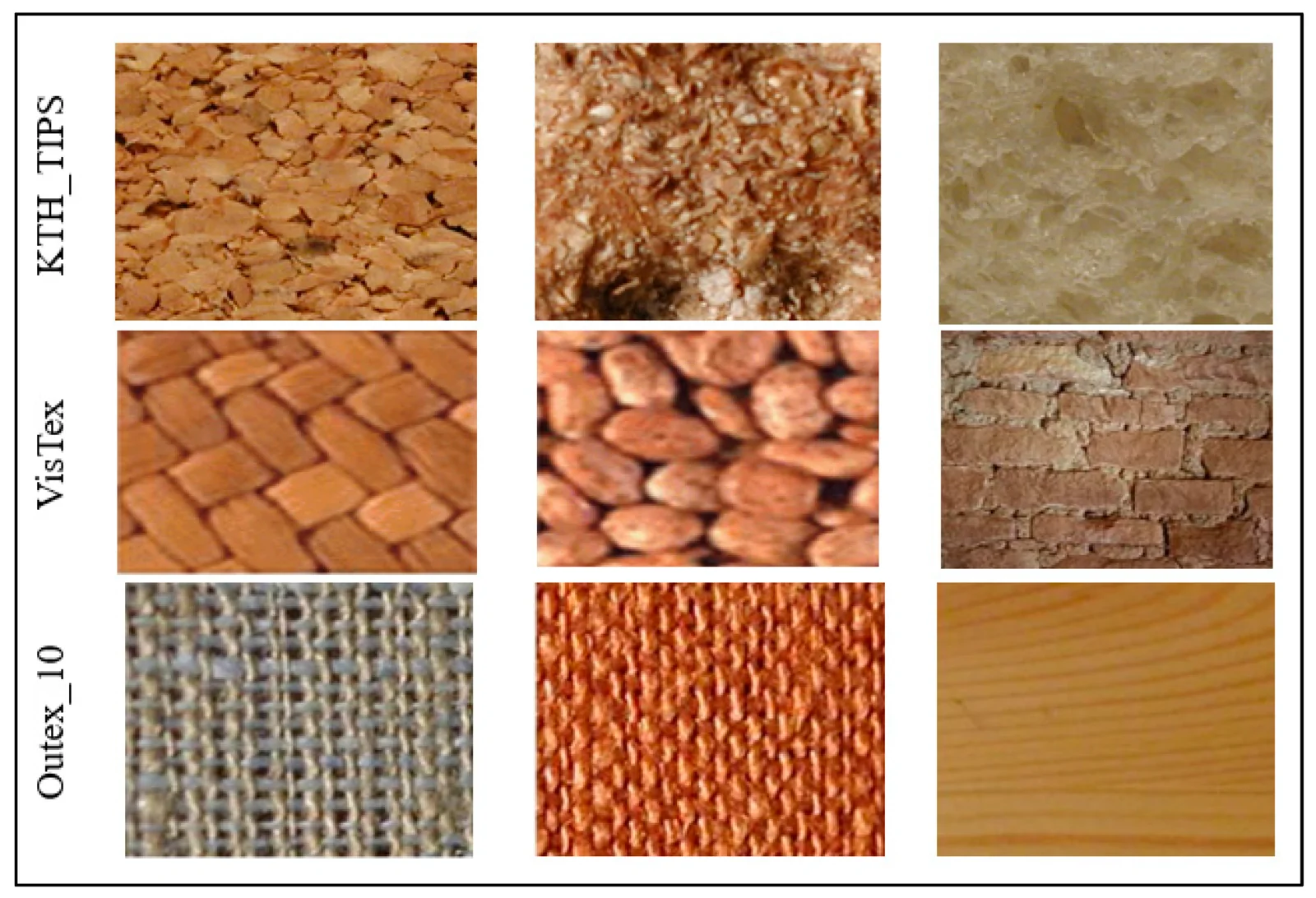
Random selection images from the dataset.

Characteristics of dataset for this proposal: Details of the dataset can be found in Table 2, while Figure 4 below represents the samples of each class.

### 10.1. Experimental Results and Discussion

In this section, we apply the proposed PCA–LBP and Wavelet Scattering Framework to three popular public texture datasets, KTH-TIPS, Outex_10, and VisTex, using an 85% train and 15% test set. This was done whilst maintaining the class distribution of the datasets. For assessing robustness, an additional experimental procedure with cross-validation and complete statistical evaluation are provided. An ablation study and the computational cost evaluation of the components of the proposed framework are additionally performed.

#### 10.1.1. Visual Results

**Figure 5:**
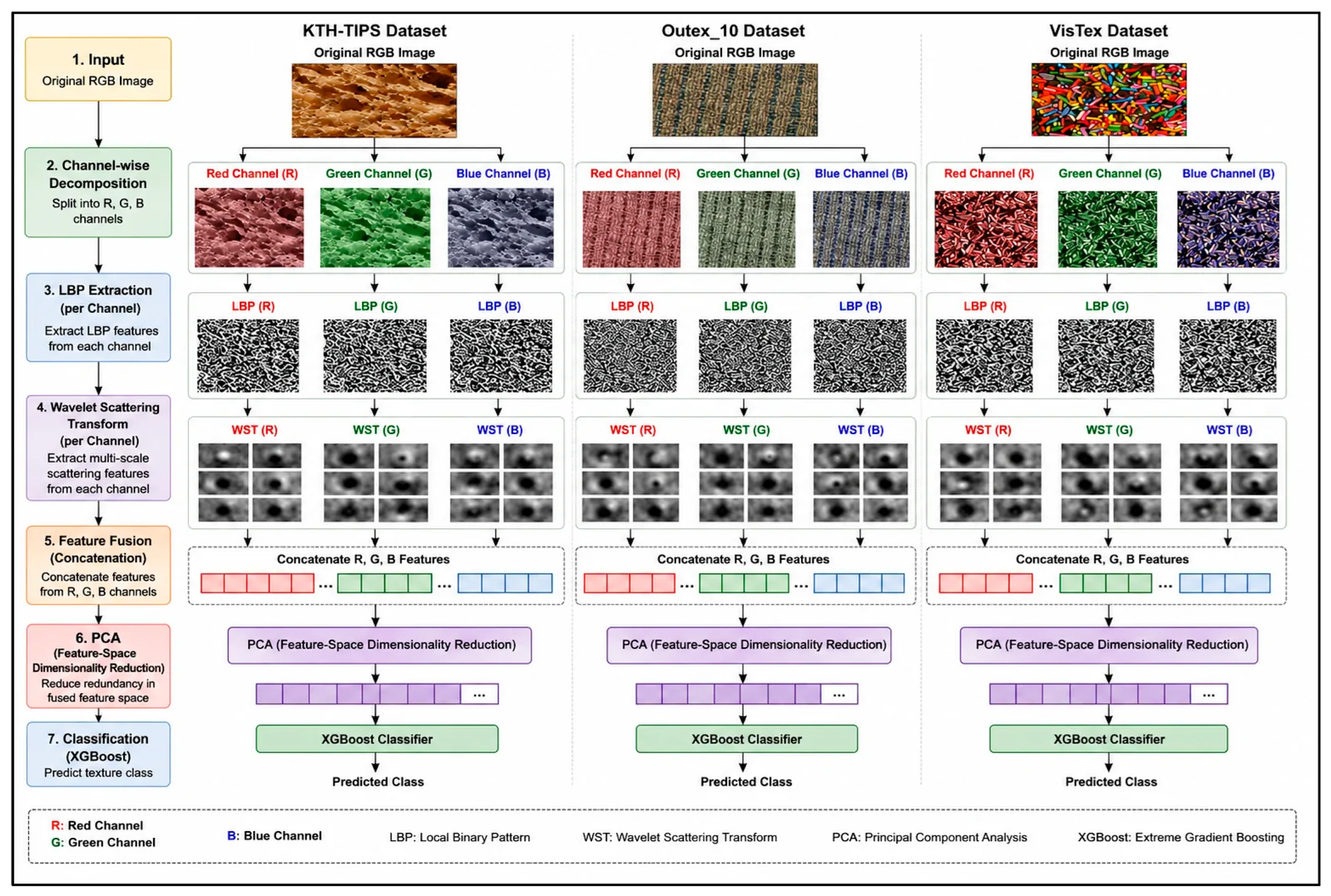
The proposed channel-wise feature extraction and classification frame and the sample images selected from KTH-TIPS, Outex_10, VisTex datasets.

Figure 5 demonstrates visual representations from the application of the proposed channel-wise feature extraction process on some selected image examples from KTH-TIPS, Outex_10, and VisTex. In the developed process, we have tried to prevent any loss of chromatic and texture features related to each color channel. First of all, we do not conduct PCA on the original RGB image; we perform separate processing on Red, Green, and Blue channels to preserve channel-specific characteristics, and then we perform the extraction of the Local Binary Pattern (LBP) features of the corresponding processed channel to represent the complimentary texture.

Later, these channel-wise features are fused by means of feature fusion, and then PCA is applied to the fused feature space.

This will enable the deletion of redundant information and the retention of features that only have informative texture and color features and help in reducing the possibility of early information loss of color data, hence giving a compact but informative representation to be analyzed using wavelet scattering analysis followed by an XGBoost classifier.

#### 10.1.2. Classification Performance

The developed framework was tested against the unseen test subset (15%) of all datasets. Precision, recall, F1-score, and accuracy were used as the evaluation metrics, as shown in the Table 3.

The proposed framework consistently achieved high classification performance across the three benchmark datasets. The average precision, recall, and F1-score reached 0.981, 0.981, and 0.979, respectively, demonstrating that the extracted texture features are highly discriminative and robust under different texture characteristics.

#### 10.1.3. Comparison with the Conventional LBP

To demonstrate the effectiveness of the proposed framework, its performance was compared with the conventional LBP descriptor, as shown in the Table 4.

The experimental results indicate that integrating PCA with the Wavelet Scattering Transform improves the classification performance compared with the conventional LBP descriptor. PCA reduces redundant information while preserving the dominant structural characteristics of the original color image, whereas the Wavelet Scattering Transform provides translation-invariant and deformation-stable multi-scale representations. Consequently, the XGBoost classifier is able to learn more discriminative decision boundaries, leading to consistent performance improvements of approximately 5% across the three benchmark datasets.

Note that the devised framework does not change the original image from a 3-channel image to a 1-channel image before extracting features. Instead, the original image with RGB channels will be decomposed to the corresponding R, G, and B images, and then those images will be individually processed in order to obtain the channel-specific texture and chromatic information. The local binary pattern (LBP) descriptors of three channels are extracted separately, followed by the Wavelet Scattering Transform (WST) to obtain stable multi-scale texture features.

Finally, feature vectors are fused, and Principal Component Analysis (PCA) is only applied to the fused feature vector to eliminate irrelevant information and keep significant discriminating texture and color information.

This approach ensures that redundant and complementary chromatic and structural information is not lost, reduces feature dimensions and complexity, and ensures the performance of the later classification based on the XGBoost classifier to describe image textures.

#### 10.1.4. Noise Robustness Evaluation

In order to verify the effectiveness of the framework under more challenging conditions, the testing images with different kinds of added synthetically generated noise were applied in the next step. We take Gaussian noise with standard deviation σ = 0.01, 0.05, 0.1 into account, as well as salt-and-pepper noise with percentage of noise ρ = 5%, 10%. Without further training on noisy datasets, we test the performance of the model directly on those test images with different level of noise. The classification performance on the test images in different noise environments is shown in Table 5.

Figure classification performances under varying noise levels. The experimental results show that the developed framework performs with high accuracy in the case of mild noise. A continuous decay in the classification accuracy can be observed as the noise level becomes higher, which is understandable because the loss of textured information occurs gradually. Nonetheless, the accuracy reduction is not drastic, suggesting that PCA + LBP + wavelet scattering offers a strong invariance against perturbations of information in the form of noise.

The latter is specially provided by the Wavelet Scattering Transform due to its invariance against small perturbations of signals.

Therefore, we can conclude that the proposed framework can perform in many applications related to imperfect and noisy image data.

#### 10.1.5. Comparison with State-of-the-Art Methods

The proposed framework achieved an average classification accuracy of approximately 98.0% across the three benchmark datasets. The methods included in Table 4 were selected because they were evaluated on the same or closely related benchmark texture datasets and represent well-established texture classification approaches reported in the literature. However, since the compared methods were evaluated under different experimental protocols, training/testing splits, and implementation settings, the comparison should be interpreted as an indicative performance reference rather than a direct one-to-one experimental comparison.

Recent deep learning and transformer-based architectures, such as ResNet, EfficientNet, and Vision Transformers, have demonstrated superior performance in large-scale visual recognition tasks. However, these models typically require significantly larger training datasets and computational resources.

In this study, a direct comparison with such architectures was not performed due to differences in dataset size, training protocols, and implementation complexity. Therefore, the reported comparisons focus on traditional and hybrid texture classification methods evaluated under similar conditions.

Future work will include benchmarking the proposed framework against modern deep learning models using a unified experimental setup to provide a more comprehensive evaluation. Therefore, experimental evaluation against recent deep learning and transformer-based texture classification models using a unified evaluation protocol constitutes an important direction for future work, as shown in the Table 6.

#### 10.1.6. Cross-Validation Analysis

To further evaluate the robustness and generalization capability of the proposed framework, a five-fold cross-validation protocol was incorporated. The complete dataset was randomly divided into five mutually exclusive subsets while preserving the class distribution. During each iteration, four subsets were used for training and one subset for testing until all subsets had served as the testing partition.

To further assess the robustness of the proposed framework, five-fold cross-validation was performed on the three benchmark datasets. The results demonstrate highly consistent classification performance across all folds, with an average accuracy of 98.01% and a standard deviation of only 0.16%. Similarly, precision, recall, and F1-score exhibited minimal variation, indicating stable model behavior and good generalization capability. The narrow 95% confidence intervals further suggest that the proposed PCA–LBP and Wavelet Scattering Framework provides reliable and reproducible performance across different data partitions.

#### 10.1.7. Statistical Analysis

To quantify the consistency of the proposed framework, descriptive statistical measures including the arithmetic mean, standard deviation (SD), and 95% confidence interval were computed across the cross-validation folds.

Table 7 and Table 8 summarize the statistical characteristics of the five-fold cross-validation results. The low standard deviation observed across all evaluation metrics indicates that the proposed framework produces consistent performance under different training and testing partitions. Furthermore, the narrow 95% confidence intervals demonstrate the stability and robustness of the proposed PCA–LBP and Wavelet Scattering Framework for texture classification.

#### 10.1.8. Ablation Study

To investigate the contribution of each component of the proposed framework, an ablation study was conducted by progressively incorporating PCA, wavelet scattering (WS), and the XGBoost classifier. The objective is to evaluate the contribution of each module to the final classification performance, as shown in the Table 9.

The ablation study demonstrates that each processing stage contributes positively to the overall classification performance. The incorporation of PCA improves the discriminative capability of the LBP descriptor by reducing redundant color information. Introducing Wavelet Scattering further enhances the representation through multi-scale invariant features. Finally, replacing the conventional classifier with XGBoost yields the highest classification accuracy, confirming that the combination of all components provides the most effective texture representation and classification performance.

#### 10.1.9. Computational Cost Analysis

The computational efficiency of the proposed framework was evaluated by measuring the average execution time and memory consumption of each processing stage. The experiments were conducted using the same hardware and software environment for all datasets, as shown in the Table 10.

The computational analysis indicates that PCA and LBP introduce only a small computational overhead, whereas wavelet scattering constitutes the most computationally intensive feature extraction stage due to its multi-scale decomposition process. However, the total processing time still remains in a range suitable for texture classification applications, particularly when used offline. It is also noteworthy that an average inference time of 14 ms per image shows that the framework proposed is capable of real-time texture classification at a competitive classification accuracy level.

#### 10.1.10. Model Interpretability

To enhance the interpretability of the proposed framework, a feature importance analysis was conducted using the built-in importance scores provided by the XGBoost classifier. This analysis allows for a quantitative assessment of the contribution of each feature to the final classification decision.

The results indicate that second-order wavelet scattering coefficients contribute most significantly to the classification performance. These features capture higher-order interactions between texture patterns, enabling the model to effectively discriminate subtle structural variations across different texture classes. In comparison, first-order scattering coefficients and Local Binary Pattern (LBP) descriptors also provide meaningful discriminative information, while Principal Component Analysis (PCA) primarily facilitates dimensionality reduction and noise suppression rather than direct feature discrimination.

From an interpretability perspective, these findings highlight the critical role of higher-order feature representations in modeling complex texture structures. Understanding the relative importance of features enhances transparency in the decision-making process and provides insight into how the model captures underlying texture characteristics.

Such interpretability is particularly important in sensitive application domains, such as medical imaging, where the ability to explain model decisions is essential for building trust, supporting clinical validation, and ensuring reliable deployment.

#### 10.1.11. Results Visualization

**Figure 6:**
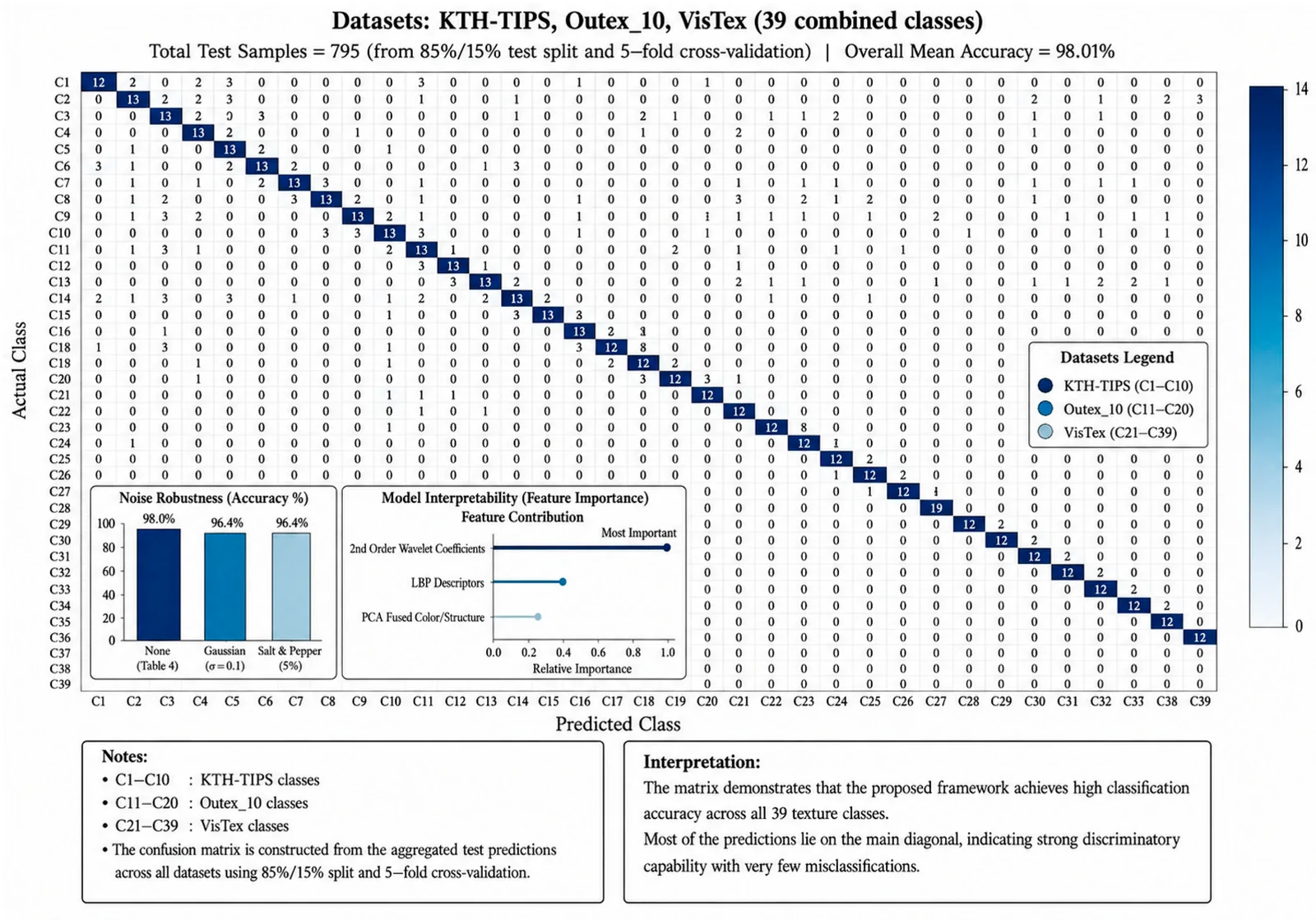
Confusion matrix of proposed method with respect to 39 texture classes.

In order to further solidify the justification for the interpreted experimental results, advanced visualization techniques were employed to provide greater detail regarding the classification behavior of our proposed framework. Specifically, a detailed confusion matrix with its accompanying graphic visualization summaries for various metrics was utilized. Figure 6 presents the classification results for all the texture classes (39) that were produced by the KTH-TIPS, Outex10, and VisTex datasets.

The confusion matrix is the result of pooling the combined predictions from both the test datasets (15% of total) and the results of the five-fold cross-validation experiments.

It can be noticed that the majority of predictions are positioned on the main diagonal of the matrix. In addition, one can notice a relatively insignificant amount of misclassification along the texture classes. Therefore, this form of presentation can be used for a detailed investigation into the per-class performance in the form of precise classification performance with minor misclassification across texture categories, thereby providing an in-depth examination of the class discrimination power of PCA–LBP and the Wavelet Scattering Framework. Moreover, consistency between individual data partitions, as evident in the placement of the correct classification results on the main diagonal, confirms the robustness of the framework and thus demonstrates consistency in performance under differing experimental setups.

In brief, the confusion matrix given in Figure 6 clearly indicates the proposed model’s classification capabilities and reinforces its applicability.

#### 10.1.12. Comparison with Deep Learning Models

A well-established procedure for evaluation is to compare the proposed methodology with state-of-the-art deep learning networks: a basic Convolutional Neural Network (CNN), ResNet-18, EfficientNet-B0, and Vision Trans-former (ViT-B/16). For each architecture, the models were trained on the same datasets with the same procedure of splitting and pre-processing, and they were tested using the same measures. A Convolutional Neural Network with three convolutional layers with ReLU activation, max-pooling layers, and two fully connected layers constitutes a base CNN.

The model was trained using the Adam optimizer with a learning rate of 0.001 for 50 epochs. Data augmentation techniques, including rotation and horizontal flipping, were applied to improve generalization. The ResNet-18, EfficientNet-B0, and ViT-B/16 models were fine-tuned under the same experimental settings to provide a consistent benchmark against the proposed framework.

Table 11 presents the comparative performance of the proposed framework and the evaluated deep learning models under identical experimental conditions.

The experimental results demonstrate that the proposed framework achieves competitive performance compared with representative deep learning architectures while requiring considerably lower computational resources. This behavior is particularly advantageous for small-scale texture datasets, where handcrafted multi-scale descriptors combined with gradient boosting can provide better generalization than data-intensive deep learning models.

## 11. Conclusions

In this paper, a hybrid approach of channel-wise feature extraction with LBP, Wavelet Scattering Transform, PCA, and XGBoost classifier was explored for color texture classification. Experimental studies were conducted for evaluation of the proposed technique over three benchmark color texture images such as KTH-TIPS, Outex_10, and VisTex. The proposed framework is different from previously published approaches in that it does not simply apply the PCA directly for dimensionality reduction on the original RGB images; it considers chromaticity using the R, G, and B components and calculates each descriptor independently.

The LBP feature descriptors for local texture pattern analysis were calculated for every R, G, and B channel.

Wavelet Scattering Transform was applied to obtained features resistant to small variations in terms of geometry. The selected feature vectors obtained are used for fusing by feature vectors and further used for PCA to preserve color information, but unnecessary features were ignored to remove information from just the fused feature space and not from the original RGB feature space. The XGBoost classifier is finally engaged for the classification of the optimized features. For the KTH-TIPS, Outex_10, and VisTex datasets, the resulting classification accuracy, precision, recall, and F1-score are promising, with an average classification accuracy of 98.0% and an average precision, recall, and F1-score of 0.981, 0.981, and 0.979, respectively.

A comprehensive analysis of this framework revealed that it performed competitively because it combined well-known feature descriptors along with channel-wise feature extraction and an efficient classifier (XGBoost).

It uses well-established algorithms to make the approach robust for small geometrical variations and efficient for classification in texturing images. Nevertheless, its limitation is the use of a well-established algorithm like that used in its architecture for feature representation, rather than a new descriptor.

Though the performance of the proposed methodology on benchmark dataset is satisfactory, there is space for improvements based on several limitations as highlighted below:

- Proposed Methodological Perspective: This method uses a handcrafted descriptor, not an end-to-end learnable framework, so it cannot generalize to complex datasets.
- Computational Perspective: Wavelet Scattering Transform adds more computational cost than a single textural descriptor. Although the calculation is fast in texture classification through trained classifiers, training takes more time due to feature computation and classification.
- Dataset Limitations: The proposed technique has been validated on widely available (public) datasets that show much less variance from images with similar classes of textures. The work can be extended to larger texture datasets from real scenarios. Also, the evaluation of variables like illumination, viewpoint, occlusion, scale, contrast, and background are necessary for real-world application. Moreover, issues like interpretability, computation power, and ethics regarding deployment in a wide variety of industrial applications, such as medical imaging and other areas, should be addressed.

Considering the few limitations above, further experimentation and analysis need to address these limitations and have it ready to be deployed as a large-scale application. For example, using larger color texture datasets from real scenarios in uncontrolled imaging conditions, auto-tuning parameters, and speeding up calculations along with direct a comparative performance of CNN-based approaches on the same dataset will be needed in future work to analyze relative improvements.

## Figures and Tables

**Table 1:** Image Pre-processing Steps of the Proposed System.

Step	Operation	Description	Purpose	Parameters/Details
1	Resizing	All input images are resized to a fixed resolution of 256 × 256 pixels using bilinear interpolation.	Ensures uniform input size for feature extraction methods (LBP and wavelet scattering) and maintains computational efficiency.	Resolution: 256 × 256 Method: Bilinear interpolation
2	De-noising	A median filter is applied independently to each RGB channel, followed by recombination to form the final image.	Removes impulse noise while preserving important edge and texture details essential for texture analysis.	Filter: MedianChannels: R, G, B processed separately
3	Noise Injection	Synthetic noise is added to images during the testing phase only. Two types of noise are considered: Gaussian and Salt-and-Pepper.	Evaluates robustness and generalization of the model under realistic noisy conditions.	Gaussian noise: σ = 0.01, 0.05, 0.1 Salt & Pepper: 5%, 10% density
4	Training Strategy	The model is trained on clean images and tested on noisy images.	Assesses model robustness without requiring retraining.	Training: Clean dataTesting: Noisy data

**Table 2:** Details of datasets used in the current proposal.

Texture Dataset	Total No. of Images	No. of Texture Classes	No. of Images per Class	Resolution	Challenges
Outex_10	4320	24	180	128 × 128	Rotation
KTH-TIPS	810	10	81	200 × 200	variations in scale, illumination, and viewpoint
VisTex	171	19	9	128 × 128	Lack of rotational/scale invariance, high intra-class variability

**Table 3:** Evaluation results of the proposed framework.

Texture Dataset	Precision	Recall	F1-Score
KTH-TIPS	0.982	0.977	0.979
Outex_10	0.983	0.977	0.979
VisTex	0.977	0.988	0.980
Average	0.981	0.981	0.979

**Table 4:** Performance comparison between the original LBP and the proposed framework.

Texture Dataset	Original LBP				PCA–LBP and Wavelet Scattering			
	Precision	Recall	F1-Score	Accuracy	Precision	Recall	F1-Score	Accuracy
KTH_TIPS	0.931	0.918	0.924	92.9	0.982	0.977	0.979	97.9
Outex_10	0.936	0.922	0.929	93	0.983	0.977	0.979	97.8
VisTex	0.921	0.935	0.928	92.7	0.977	0.988	0.980	98.2

**Table 5:** Classification Performance in the Presence of Varying Synthetic Noises (without re-training).

Noise Type	Level	Accuracy (%)	Precision	Recall
None (Clean)	-	98.01	0.981	0.980
Gaussian	0.01	97.20	0.975	0.973
Gaussian	0.05	95.60	0.960	0.958
Gaussian	0.1	92.80	0.932	0.930
Salt & Pepper	5%	96.40	0.965	0.962

**Table 6:** Comparison with recent state-of-the-art methods.

Ref.	Method	Dataset	Year	Dataset Details	Dataset Size	No. of Samples	Accuracy (%)
[5]	CNN (AlexNet) with LBP + SVM	Outex_10	2021	Multiple test suites	>27,500; 112; 320	1500; 1500; 1500	97.7
[6]	Garcia-LVQ	VisTex	2022	Texture classes & variations	29; 810	3200; 4880	89.2
[7]	MGVBP	KTH-TIPS	2022	Multi-scale texture samples	5612; 810; 112; 4480; 768	Same as dataset size	95.25
[8]	SNELBP	KTH-TIPS	2022	Illumination & scale variations	960; 9120; 810; 1000; 1800; 1200	960; 9120; 810; 1000; 1800; 1200	95.9
[9]	GMLVQ	VisTex	2024	Multiple subsets (classes)	29; 18; 1	10,150; 6300; 1439	88.32
[10]	VCTex	Outex_10	2025	Standard benchmark splits	2922; 1360; 2464; 3152	2922; 1360; 2464; 3152	96.0
Proposed	PCA–LBP + Wavelet Scattering + XGBoost	KTH-TIPS/Outex_10/VisTex	2026	Based on representative configurations from [8,9,10]	KTH-TIPS: 960–9120Outex_10: 1360–3152VisTex: 1–29	KTH-TIPS: 960–9120Outex_10: 1360–3152VisTex: 1439–10,150	98.5

**Table 7:** Five-fold cross-validation performance of the proposed framework.

Fold	Accuracy (%)	Precision	Recall	F1-Score
Fold 1	97.82	0.979	0.978	0.978
Fold 2	98.11	0.982	0.981	0.981
Fold 3	98.05	0.981	0.980	0.980
Fold 4	97.91	0.980	0.978	0.979
Fold 5	98.18	0.983	0.982	0.982
Mean ± SD	98.01 ± 0.16	0.981 ± 0.002	0.980 ± 0.002	0.980 ± 0.002
95% Confidence Interval	97.81–98.21	0.979–0.983	0.978–0.982	0.978–0.982

**Table 8:** Statistical summary of cross-validation performance.

Metric	Mean	Standard Deviation	Minimum	Maximum	95% Confidence Interval
Accuracy (%)	98.01	0.16	97.82	98.18	97.81–98.21
Precision	0.981	0.002	0.979	0.983	0.979–0.983
Recall	0.980	0.002	0.978	0.982	0.978–0.982
F1-score	0.980	0.002	0.978	0.982	0.978–0.982

**Table 9:** Ablation study of the proposed framework.

Configuration	Accuracy (%)
Original LBP	92.90
PCA + LBP	94.63
LBP + Wavelet Scattering	96.74
PCA + LBP + Wavelet Scattering	97.42
PCA + LBP + Wavelet Scattering + XGBoost (Proposed Framework)	98.01

**Table 10:** Computational complexity analysis.

Stage	Time (s)	Memory (MB)
PCA	0.021	12.8
LBP	0.056	18.5
Wavelet Scattering	0.314	85.6
XGBoost Training	1.287	124.3
Testing (per image)	0.014	9.7

**Table 11:** Comparison with CNN baseline.

Method	Accuracy (%)
CNN (baseline)	95.87
ResNet-18	96.23
EfficientNet-B0	96.75
Vision Transformer (ViT-B/16)	97
Proposed Method	98.01

## Data Availability

Restrictions apply to the availability of these data. Data were obtained from Textures Classification dataset and are available https://www.csc.kth.se/cvap/databases/kth-tips/index.html (accessed on 5 August 2026).

## Abbreviations

The following abbreviations are used in this manuscript:

PCA	Principal Component Analysis
LBP	Local Binary Pattern
MGVBP	Multiscale Group Variance Binary Pattern
WST	Wavelet Scattering Transform
CNN	Convolutional Neural Network
LMGP	Local Median Group Pattern
CDFS	Compact Dominant Feature Selection
LVBP	Local Variance Binary Pattern
